# The Adolescent Knee Pain (AK-Pain) prognostic tool: protocol for a prospective cohort study

**DOI:** 10.12688/f1000research.21740.2

**Published:** 2020-02-21

**Authors:** Alessandro Andreucci, Sinead Holden, Martin Bach Jensen, Michael Skovdal Rathleff

**Affiliations:** 1 Center for General Practice at Aalborg University, Aalborg University, Aalborg, North Denmark Region, 9220, Denmark; 2 Center for Sensory-Motor Interaction (SMI), Department of Health Science and Technology, Faculty of Medicine, Aalborg University, Aalborg, North Denmark Region, 9220, Denmark

**Keywords:** Prognosis, Prognostic factor, Adolescent, Knee pain

## Abstract

**Background: **One in three children and adolescents experience knee pain. Approximately one in two adolescents with knee pain will continue to experience pain even five years later and have low quality of life. The general practitioner (GP) is the first point of contact for children and adolescents with knee pain in Denmark. There is a variety of treatments being delivered in general practice, despite similar symptoms and patients’ characteristics. This suggests a need to support the GPs in identifying those at high risk of a poor outcome early on, in order to better allocate resources. The aim of this study is to develop a user-friendly prognostic tool to support GPs’ management of children and adolescents’ knee pain.

**Methods: **A preliminary set of items in the prognostic tool were identified using systematic reviews and meta-analysis of individual participant data. Following feedback from GPs and children and adolescents on the content and understanding, the tool was piloted and implemented in general practice. A cohort of approximately 300 children and adolescents (age 8-19 years old) is being recruited from general practices (recruitment period, July 2019 – June 2020). Clinically meaningful risk groups (e.g. low/medium/high) for the recurrence/persistence of knee pain (at 3 and 6 months) will be identified.

**Discussion: **If successful, this prognostic tool will allow GPs to gain insights into the likely prognosis of adolescents with knee pain and subsequently provide the first building blocks towards stratified care, where treatments will be matched to the patients’ prognostic profile. This has the potential to improve the recovery of children and adolescents from knee pain, to improve the allocation of resources in primary care, and to avoid the decline in physical activity and potential associated health and social consequences due to adolescent knee pain.

**Registration: **Registered with ClinicalTrials.gov on 24 June 2019 (ID NCT03995771).

## Background

One in three adolescents experience knee pain ^1^. Knee pain is associated with low quality of life and lower sporting ability compared to adolescents without knee pain ^1^. In addition to the impact on the individual adolescents, knee pain has an impact on their family ^2^ and an economic impact, due to both direct (e.g. primary care visits, community services use, medication use) and indirect (e.g. parental productivity work loss and days off work) costs ^3, 4^. Adolescent knee pain (AKP) was once thought to be innocuous and self-limiting, but new data has challenged this assumption ^5^. A recent prospective cohort study demonstrated that 40% of adolescents with knee pain still experienced knee pain even after five years ^5^. Knee pain is linked to both health and social consequences ^5, 6^. Children and adolescents with knee pain are likely to reduce their sport participation, which may have implications for overall health in later life (e.g. higher adiposity, impaired sleep) ^1, 7– 10^. For some adolescents it results in school absence ^8, 11^ and for one in seven it affects their choice of job or career ^5, 12^.

While there is a large body of knowledge on adult knee pain ^13– 15^, less is known in children and adolescents ^7, 16^. Potential prognostic factors for a poor outcome in AKP include female sex, high leisure time sport participation, low health-related quality of life, high baseline frequency of knee pain ^6^. These preliminary prognostic factors have been identified from single studies and have never been replicated in independent cohorts. Therefore, there is a need to further test and replicate these prognostic factors in other studies in order to confirm this preliminary evidence, also in contexts such as primary care.

Children and adolescents with knee pain commonly consult their general practitioner (GP), who is their first point of contact and who discuss with them and their caregivers the different treatment options (e.g. referral to a specialist, education on how to manage knee pain, or exercises). This is largely based on clinical experience as there is a lack of clinical practice guidelines and original research on the management of AKP. This may result in heterogenous care, unnecessary over-medicalization ^17, 18^ and large differences in treatments, despite presenting with similar symptoms and characteristics.

One potential option to support clinical decision making is to develop decision aids such as prognostic tools. These tools often consist of items with prognostic value that can be asked during the clinical consultation or completed prior to the consultation, and can be used to stratify patients depending on their prognosis ^19^ and subsequently the best targeted treatment based on the prognostic profile can be offered the patient. In the case of a child or adolescent with knee pain, different treatments or recommendations (e.g. short education session, modification of physical activity levels, exercise, use of painkillers, referral to a specialist) might be provided depending on the risk of a poor prognosis. Examples of prognostic tools that have already been developed include the Keele STarT Back Screening Tool (SBST) for lower back pain in adults ^20^ or the Pediatric Pain Screening Tool (PPST) for general pediatric pain ^19^. However, a prognostic tool to be used specifically for the prognosis of AKP in primary care has not been developed yet. The development of a prognostic tool for AKP would fill this gap and provide supporting information to guide GPs in their clinical decision towards a stratified care based on the category of risk for AKP (derived from the patients’ individual characteristics).

## Methods

### Aim of the study

The aim of this study is to develop and test a prognostic tool for AKP to be used in general practice.

### Study design

This study is designed as a prospective cohort study and the protocol follow The Recommendations for Interventional Trials (SPIRIT) 2013; the completed checklist can be accessed at the Harvard Dataverse online repository ^21^.

### Study setting

Participants will be recruited from general practices in Denmark, with data collected from July 2019 until June 2020.

### Recruitment strategy

The recruitment strategy for the project has been developed together with experienced GPs and researchers within the field of general practice to ensure feasibility of recruitment and high generalisability. The recruitment includes two stages; the recruitment of GPs and the recruitment of participants.

### Eligibility and recruitment of GPs

GPs working in the North Denmark Region and Odense area in Denmark are eligible for inclusion. These areas were chosen for logistic reasons, but more regions (e.g. Central Denmark Region, Copenhagen area) may be added if recruitment is slower than anticipated. To obtain enough GPs involved in the study, efforts will be done to comply with the Solberg’s seven R-factors for recruiting medical groups for research (i.e. relationship, reputation, requirements, reward, reciprocity, resolute behaviour and respect) ^22^. First, contact will be made with GPs in order to ask them their availability to join the research. The first contact will be brief, but informative. Second, introductory meetings will be held with GPs and their staff (i.e. secretaries and nurses) to confer the importance, contents and goals of the study, information regarding the participants’ eligibility criteria and to plan the recruitment of participants. After the beginning of participant recruitment, regular meetings with the GPs and their staff will be held during the follow-up in order to monitor the recruitment rate of participants and support them in recruitment (e.g. if changes are needed to the strategy used at their clinic).

### Eligibility and recruitment of participants

Children and adolescents who consult their GP because of knee pain (of both traumatic and non-traumatic origin) during a period of recruitment of at least 6 months (start in July 2019) are eligible for inclusion. Children and adolescents will have to be between 8 and 19 years old. The age of 8 is considered to be the lowest age for the children to be able to complete a pain questionnaire or a pain chart without adult guidance ^23^ (provided that the questions are properly worded by taking into account the age-related cognitive abilities ^24, 25^), and the age of 19 is defined as the upper limit for the period of adolescence by the World Health Organization ^26^. The following exclusion criteria will be assessed and applied by the person in charge of recruiting the participants (i.e. either the GP or a member of the staff):

- Age below 8 years old or over 19 years old
- Consultation for musculoskeletal pain only in a body region different from the knee
- Pain originated from specific non-musculoskeletal conditions (e.g. cancer, infections)
- Child is vulnerable (e.g. he/she has experienced a recent trauma and the distress may have an impact on the self-report making it not valid) ^24^
- Inability to take part to the study because of inability to understand the questionnaire (i.e. participants who have issues with Danish language or have learning difficulties)

Participants will be recruited from general practices on the basis of consultation for knee pain. In Denmark, consultations with the GP are booked beforehand, which allows the GP and the GP’s staff to know in advance what the patient is consulting for. The study will be introduced to the caregivers and children before the consultation by a person working at the general practice (e.g. the GP/nurse/secretary). If patients meet the inclusion criteria for being eligible into the study (e.g. patient aged between 8 and 19 years old who consult for knee pain) and agree to participate, they will be provided with the study material (i.e. prognostic tool to be completed). However, if the GP was made aware of the issue of the presence of knee pain during a consultation regarding other health issues, the questionnaire will be completed after the consultation. In the primary stages of the study the person in charge of recruiting participants will be assisted in recruitment by the primary investigator of this study (A.A.) or by a research assistant who will attend the participating general practices.

Complementary recruitment strategies that might be applied to maximize the recruitment rate are the advertisement of the study through social media (e.g. Facebook, Twitter, Snapchat) and through explicative posters displayed at the participating general practices. In this case, it will be screened that participants have been seen by their GP within the last week and knee pain was part of the consultation. These complementary strategies will be applied if less than approximately 100 participants in 3 months will be recruited.

To encourage participation, children and adolescents will be offered two cinema tickets to take part to the study. The first ticket will be given at baseline, when children and adolescents decide to take part in the study and return the questionnaire. The second ticket will be given when the participants have completed the follow-up (e.g. provided information at both the 3-month and 6-month follow-up points).

### Data collection

Data will be collected through a questionnaire delivered at the general practice to children and adolescents who consult for knee pain (available as *Extended data* ^27^). The questionnaire will be either paper-based or collected with the support of a tablet through a link to the REDcap web application for online surveys ^28^ depending on the GPs’ preference for data collection. A further recruitment through social media might be applied to complement the baseline collection of data, with data collected through a questionnaire home-mailed or delivered with an e-mail with a link to the web application for online surveys.

Outcomes will be collected by questionnaires. The adolescent can choose to do this self-reported or caregiver-reported (through an e-mail with a link to the web application for online surveys or text message) at follow-up (two time points: 3-month and 6-month follow-up). The questionnaires will include questions about pain characteristics (e.g. severity of pain, period free of knee pain, disability and activity limitations due to pain ^29, 30^) taken from previously validated pain questionnaires or pain scales. The questionnaire also includes a question about who the person replying to the questionnaire is (i.e. child alone/caregiver/child together with the caregiver). In order to limit loss to follow-up, an e-mail/text message reminder will be sent to participants if they do not complete the follow-up questionnaire within one week from the day when they are supposed to reply (i.e. 3-month and 6-month follow-up). A second reminder will be sent one week after the first reminder if they still will not have completed the follow-up questionnaire. Reasons for loss to follow-up will be assessed by contacting through a phone call/e-mail/text message those participants who do not reply to 3 consecutive follow-up reminders and asking about reasons for leaving the study.

### Recruitment and retainment of participants

**Figure 1.**
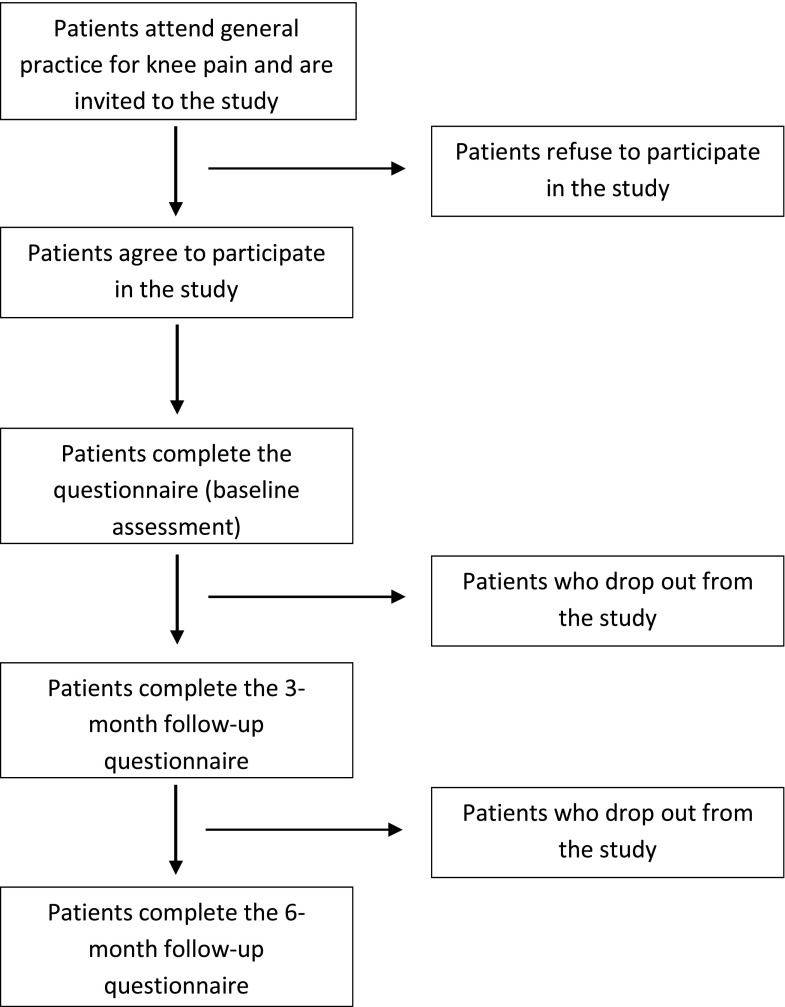
Recruitment and retainment of participants in the study.

The process of recruitment and retainment of participants in the study (data collection start date: July 2019 – end date: June 2020) is described in the following flow diagram ( Figure 1).

### Development of the tool

Prognostic factors for knee pain in children and adolescents that can be measured in the context of general practice were identified from a review of the current literature on the topic ^31^. The review included 26 prospective studies, of which 4 focused on knee pain. These 4 studies included schoolchildren 12–19 years old from Denmark (3 studies) and 10–12 years old from Finland. Initially, the most important domains for the prognosis of knee pain and the specific items to be included in the tool were selected based on the review but also from the strength of association from previous studies identified within the literature and from meta-analysis of individual participant data. Prognostic factors for knee pain identified within the review ^31^ were increasing age, pain frequency, practicing sport more than 2 times/week and low quality of life (second question of item 9 within the tool). Other factors that were identified from the wider literature and meta-analysis of individual participant data (PROSPERO ID CRD42019116861) were knee pain characteristics (pain duration, traumatic/non-traumatic pain onset, limitations in daily activities due to knee pain, presence of knee pain in one knee or both knees), presence of pain in other body sites, gender, sleep, smoking, psychological factors, parental history of pain. During this stage, great emphasis was given to include only the most necessary factors considering the ultimate use of the prognostic tool (i.e. it should be used in a reasonable time of a consultation within general practice). Items relative to the specific prognostic factors were initially selected from validated scales when possible (e.g. regarding pain characteristics, limitation in daily activities, sport participation, psychological factors, sleep), or from previous studies. When multiple items within a scale or multiple scales were available for a prognostic factor, the relevant literature on the topic was identified and discussed at meetings with GPs and staff working at the Center of General Practice at Aalborg University in order to select the most appropriate items within the possible options. For example, the sleep item was selected from the self-reported Pediatric Quality of Life Inventory, one question of the psychological factor item from the Pain Catastrophizing Scale for Children, one question of the psychological factor item and the item relative to limitations in daily activities from the EuroQol Five-Dimensional Questionnaire Youth version. Following previous tools developed for pain in children and adolescents ^19, 29, 32^, items were properly worded for the age and properly framed with respect to the response options (e.g. direction, time intervals, avoiding double-barrelled items). A process of forward-backward translation of each item from Danish to English was applied to ensure that the items worded in Danish were conceptually equivalent to the items worded in English which were selected from previous studies or validated tools.

### Study piloting

**Figure 2.**
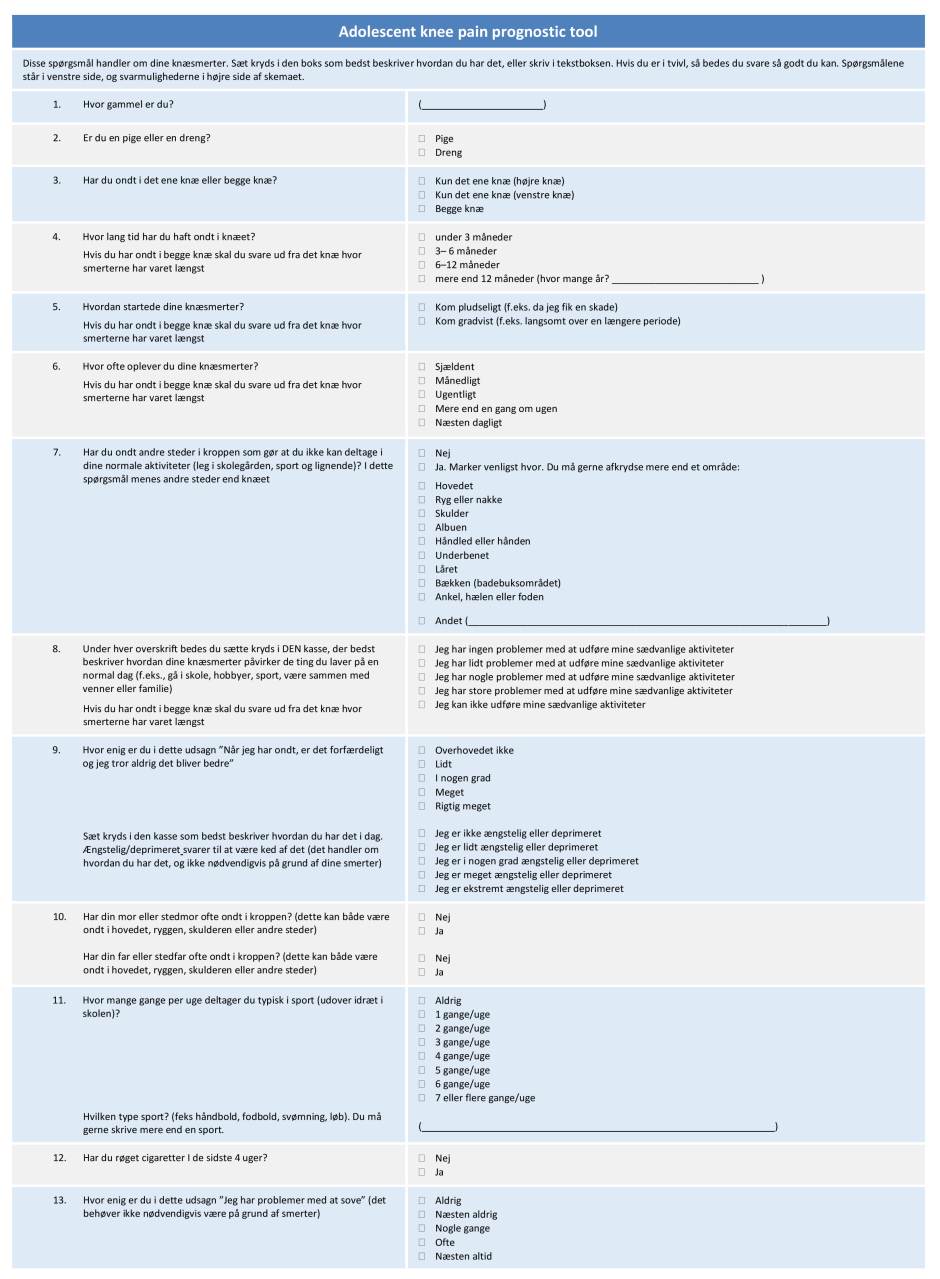
Adolescent Knee Pain prognostic tool.

This project included a stage where the prognostic tool was piloted, which included a development, testing and implementation stage. After the initial development stage described above, the prognostic tool was tested with volunteer participants (n = 14) recruited through advertisement of the study on Facebook. Children and adolescents who were interested in the study (or their caregivers) contacted the primary investigator of this study (A.A.) for taking part in the test, and a date was arranged for testing the tool and carrying out cognitive interviews with a research assistant (T.S.). The test and cognitive interviews were carried out either in person at the Center for General Practice at Aalborg University or through Skype. The initial version of the tool was delivered to participants and asked to be completed, and the time needed for completion was assessed. After completion of the tool, cognitive interviews were carried out to assess the appropriateness, comprehensibility, wording and potential lack of items relating to the prognosis of knee pain, as previously done for other tools that evaluated pain status in children and adolescents ^29, 32– 34^. The questionnaire for cognitive interviews is available as *Extended data* ^35^. The aim was to improve the face, construct and content validity of the tool at this stage. This is especially important considering that a worse outcome can result if there is a lack of communication between the GP and the child about the knee pain characteristics and the factors related to knee pain assessed within the tool ^36^. After receiving feedback through cognitive interviews, the prognostic tool was implemented to reach the optimal final version to be used in the data collection stage ( Figure 2; also available as *Extended data* ^27^ together with the English version). Participants were given a cinema ticket as a reward for participating in the cognitive interviews.

### Stability of the tool

A pilot study to assess the stability of the prognostic tool ^33^ in children and adolescents pre- and post- consultation was carried out. Children and adolescents who consulted primary care and their caregivers were given the prognostic tool (together with the informed consent) to be completed in the waiting room of the general practice before the consultation. Subsequently, after the consultation with the general practitioner, children and their caregivers were asked to complete the prognostic tool again in order to assess the stability of the tool parameters and the general practitioner’s influence on the parameters (e.g. pain perception and psychological factors) assessed with the tool. Differences in reporting were assessed by means of K-statistics for categorical variables and intra-class correlation coefficient for continuous variable (e.g. age). Results, which are available as *Extended data* ^37^, showed K-statistics values above 0.80 (range from 0.66 to 1) for most items, showing good stability. Only the item relative to helplessness had a value below 0.70 (K-statistics = 0.66), which is considered the minimum standard for reliability ^33^.

### Outcome

The outcome measure will be the recurrence/persistence of activity-limiting knee pain (i.e. defined as participants reporting yes to having pain that is limiting activities in the same knee) at follow-up ^30^. Participants will be asked about continuity of their knee pain (i.e. “do you still have knee pain?” and, if they reply “no”, “when did your stop having knee pain?”), to enable the distinction between recurrence (on/off knee pain episodes between baseline and follow-up) and persistence (continuous knee pain from baseline to follow-up) of knee pain. The primary end-point that will be collected is the recurrence/persistence of activity-limiting knee pain at 3-month follow-up, while the secondary end-point is the recurrence/persistence of activity-limiting knee pain at 6-month follow-up

In addition, previous studies have shown an effect of the treatment received on the change in risk group for the recurrence/persistence of pediatric pain ^38^ and on the change in pain and function ^39^. Therefore, an additional outcome measure that will be assessed include the treatment effectiveness on the recurrence/persistence of knee pain.

### Statistics

A statistical analysis plan for the development of the final prognostic tool prior to recruitment has been developed. The analysis plan includes the following stages:

1. Descriptive analysis of the collected data, with results presented as means with SDs and as percentages.
2. Assessment of potential floor and ceiling effects of the items included in the prognostic tool. This will be done by checking that for those items that represent an ordinal or categorical variable with more than two potential response categories, the responses given are not skewed towards the top or bottom extreme of the scales (e.g. a ceiling or floor effect is present if >15% of the respondents report the lowest/highest score of the scale ^30, 33, 40^).
3. We will estimate the knee pain prognosis (i.e. recurrence/persistence of knee pain, dichotomous outcome) at 3-month and 6-month follow-up by means of multiple logistic regression to estimate ORs and 95% confidence intervals for each item included in the tool. This allows to assess the independent effects of each item and will inform on which factors are most related to the prognosis of knee pain (only the items that will show a statistically significant contribution to the model will be selected). This will also provide an insight on the scores of the prognostic tool (both overall and for subscales) to be applied for the creation of the initial risk groups. Alternatively, the RR will be estimated if another linear model analysis will be carried out. In addition, a potential option is to apply different weights to the items based on the strength of association.
4. Discriminant validity of the prognostic score will be assessed by using receiver operating characteristics (ROC) curves and by calculating the area under the curve (AUC) for the overall score and subscales of the prognostic tool.
5. Data from this sample will be used for the creation of risk groups for the recurrence/persistence of knee pain on the basis of cut-off scores identified using the ROC curves. Weights based on the strength of association identified with the multiple logistic regression might be applied. The initial idea is to have two or three risk groups (e.g. low/medium/high), which have to be clinically meaningful. More importance will be given to the sensitivity of the tool over the specificity. This means that in the presence of different cut-off scores for the inclusion of patients in the high-risk group, the cut-off that will allow to identify the majority of those with a bad prognostic outcome will be chosen. This will be done to avoid the misclassification of patients at high-risk in the medium or low-risk group.
6. Assessment of the predictive ability of the risk groups defined at baseline by calculating the sensitivity, specificity and negative and positive likelihood ratios (LRs) against the primary and secondary outcome (i.e. 3-month and 6-month recurrence/persistence of knee pain; disability/activities limitation due to knee pain).
7. Assessment of the potential influence of non-modifiable patients’ characteristics on the predictive ability of the risk groups defined at baseline by stratifying the former analysis by age groups, sex and traumatic/non-traumatic onset.

### Sample size

A sample size of minimum 300 participants from at least 20 general practices for the development of the tool has been estimated. This estimate was based on the following factors; the sample size required for the development of other prognostic tools ^19, 20^, the annual consultation prevalence for knee pain in children and adolescents in general practice, the size of general practices, the number of items in the tool and the rule of thumb of at least 10 events for variable (or items within the prognostic tool) ^33^.

Previous studies have shown an annual consultation prevalence of 104–200 per 10,000 registered persons in children aged 3 to 19 years old ^41– 43^. Several potential scenarios about participants’ recruitment were hypothesized by considering the lowest and the highest annual consultation prevalence. These scenarios were calculated on a conservative estimate of 30% study participation rate. This is a worst-case scenario, and this approach was taken in order to have a safe recruitment that will provide enough children and adolescents for the development of the tool. These calculations resulted in an estimate of at least 20 general practices needed for recruiting the participants to the study (the estimate changes depending on the annual consultation prevalence considered and the size of the general practices; full calculations are available on request to the authors). In addition, if the recruitment from general practices will provide a low number of participants, a complementary recruitment through social media will be performed in order to achieve a total sample size of at least 300 children. In this case, sensitivity analysis would be performed to check for any potential difference in characteristics between the sample recruited through general practices and social media.

### Data completeness, quality and security

The participant submitted responses will be automatically registered in a database using the REDCap. Handling of data will comply to the General Data Protection Regulation and the concomitant local data handling instructions for Center of General Practice at Aalborg University. Data will be stored at a server at Aalborg University, this will ensure a safe and legal handling of data. The accuracy of the data will be checked through screening of data outliers and potentially “wrong” or “strange” data will be identified and corrected. In order to obtain a “full analysis set” for the project, participants will have to provide data from baseline through the 3-month and 6-month follow-up, which will allow to estimate the short-term and long-term knee pain prognosis. Data completeness (i.e. completion and accuracy of data forms) will be monitored and actions will be taken to overcome potential problems such as missing data ^44^. In case of baseline missing data, the missing observation will be replaced by means of an imputation process (e.g. multiple imputation by chained equation) depending on the number of missing observations (i.e. multiple imputation is usually performed when the percentage of missing data is low). A sensitivity analysis will also be carried out in order to compare results between the analysis carried out on the dataset with missing observation (complete-case analysis) and the multiple imputed dataset (multiple imputation analysis). A backup copying of the dataset will be performed daily.

### Access to data

The final dataset will be accessed by A.A., M.S.R., M.B.J. and S.H..

### Protocol amendments

Any future protocol amendments or changes will be made publicly visible in the clinical trial registration, and clearly described in the subsequent reporting of the results.

### Dissemination

The present study will provide data on the prognosis for knee pain in children and adolescents who present to primary care. In addition, this study will provide data on the usability of a prognostic tool to allocate children and adolescents to a category of risk for knee pain recurrence or persistence and consequently provide them with the best targeted treatment. The study results will be disseminated at scientific conferences and through appropriate scientific journals. General practitioners, children and caregivers participating into the study will be regularly provided with feedback about the ongoing study as well. ****

All authors of this current paper (AA, SH, MBJ, MSR) will be involved in the production of manuscripts originating from this study.

### Ethics approval and consent to participate

Ethic approval for the study and for the pilot study was seek by sending an enquiry to the Scientific Ethics Committee for Region North Jutland, together with a brief description of the study. The response obtained from the Scientific Ethics Committee stated that ethical approval was not needed, as the study implied the use of a questionnaire survey and did not imply any type of intervention on participants. Written informed consent will be obtained by the adolescents if aged 15 years old or more, otherwise from the caregivers. Participants who will become 15 years old during the transition from baseline to follow-up will be asked to provide consent themselves when contacted at follow-up.

## Discussion

The objective of this study is to develop a user-friendly prognostic tool (Adolescent Knee Pain prognostic tool), which will be the first one to be used to support the GPs’ management of AKP. Within this study, the preliminary prognostic factors for AKP identified in the literature and included in the initial version of the tool will be tested. Those prognostic factors that will prove to be independently significantly associated with a poor AKP prognosis and will contribute to the prognostic model will be included in the final version of the tool. The tool will enable the identification of different subgroups of patients who seek primary care for AKP according to their risk of recurrence or persistence of knee pain at 3-month and 6-month follow-up.

### Limitations

A limitation is that it might be argued that specific knee pain conditions (e.g. patellofemoral pain, Osgood-Schlatter) might be characterized by different prognostic courses. However, the accurate diagnosis of specific knee pain conditions in the primary care context might be challenging, and this tool was conceived for enquiring general questions regarding AKP. Second, this tool might not be applicable to health-care systems of countries where there might be a different categorization of primary care and secondary care or where GPs might not be the sole gatekeeper of primary care provision ^45^. Third, there might be difficulties in recruiting 300 participants with AKP from general practices, and although alternative recruitment strategies have been planned (i.e. through social media), this might produce selection bias ^46^.

### Strengths

First, primary care is the place where the majority of health care is delivered ^47^, and consequently the development of a tool to be used within this setting has the potential to have a significant impact in real-life.

Second, the prognostic tool includes items about factors that are specific to the AKP prognosis (e.g. pain duration, knee pain frequency) and therefore would be more sensitive than other more general pain tools. This is important considering that misclassification of patients might potentially lead to undertreat those misclassified as low-risk and overtreat those misclassified as high-risk ^48^.

Third, the subgroup of patients who refer to primary care is usually characterized by a different severity of symptoms compared to general population, second or tertiary care samples, as proposed by the iceberg theory of disease ^49– 51^. Hence, this research has the opportunity to provide information on the predictive ability of a prognostic tool in primary care compared to studies carried out within other care settings (e.g. the PPST was validated in tertiary care settings ^19, 52^), as it has previously been observed a difference in the efficacy of risk prediction potentially because of differences in patients case mix ^53^.

Fourth, this prognostic tool is short (only 13 prognostic factors assessed overall) and quick to use (tests during the piloting of the tool showed that on average approximately three minutes and a half are needed to complete the tool) and includes factors that can be easily collected during a consultation with a GP. Finally, the tool is easy to be delivered and properly worded to be understandable by children and adolescents, as it was implemented following their feedback during cognitive interviews.

### Use of the tool for providing stratified care

The use of this tool can potentially improve the understanding of the AKP prognosis and identify specific categories of risk of a poor prognosis. However, the care needed will differ among patients with AKP. Some of them will only need conservative management (e.g. education on how to manage knee pain, modification or avoidance of physical activity), while others will need a referral to a specialist (e.g. a physiotherapist, a rheumatologist). If it will prove to perform adequately (i.e. in terms of sensitivity, specificity, positive and negative likelihood ratio), this tool will inform on the likely prognosis of AKP and potentially guide the GPs in providing a targeted stratified care according to the risk of recurrence or persistence of AKP at 3-month and 6-month follow-up. The use of this tool can potentially significantly change the use of resources and increase the primary care efficiency by allocating resources to those who need them most. The development of this tool fits within a wider research program, which overall aim is to provide a stratified approach to primary care management of child and adolescent knee pain that can result in clinical and economic benefits compared with current best practice. Therefore, future perspectives include the use of this tool in a randomized controlled trial, which will investigate whether subgrouping patients using the tool, combined with targeted treatment, is more clinically effective (i.e. it will reduce long- term disability from knee pain) and cost-effective compared to best current care. In addition, there is scope for performing future qualitative studies to assess the GPs´ behavioral change when using the tool (e.g. changes in referral to physical therapy, diagnostic tests and medication prescriptions).

## Trial status

Name of registry: ClinicalTrials.gov

Registration number: NCT03995771

Date of registration: 24/06/2019

Date of start of recruitment: 01/07/2019

Expected date of recruitment conclusion: 30/06/2020

Trial URL: https://clinicaltrials.gov/ct2/show/NCT03995771?term=NCT03995771&rank=1.

## Data availability

### Underlying data

No underlying data are associated with this article.

### Extended data

Harvard Dataverse: Dataset to test the stability of the prognostic tool. https://doi.org/10.7910/DVN/SMQRKA ^37^.

This project contains the dataset generated when testing the stability of the tool.

Harvard Dataverse: Questionnaire for cognitive interviews. https://doi.org/10.7910/DVN/UT1MGD ^35^.

This project contains the Danish- and English-language questionnaire for cognitive interviews.

Harvard Dataverse: Final questionnaire. https://doi.org/10.7910/DVN/QKWOOT ^27^.

This project contains the final Danish- and English-language questionnaire for data collection.

### Reporting guidelines

Harvard Dataverse: SPIRIT checklist for ‘The Adolescent Knee Pain (AK-Pain) prognostic tool: protocol for a prospective cohort study’. https://doi.org/10.7910/DVN/SIO5WG ^21^.

Extended data and completed reporting guidelines checklist are available under the terms of the Creative Commons Zero “No rights reserved” data waiver (CC0 1.0 Public domain dedication).
