# Supplementary material for: The Adolescent Knee Pain (AK-Pain) prognostic tool: protocol for a prospective cohort study
Source: F1000Res. 2020 Feb 21;8:2148. Originally published 2019 Dec 30. [Version 2] doi: 10.12688/f1000research.21740.2 (PMC7194341; doi:10.12688/f1000research.21740.2)
Supplement: Peer review history

# Peer review: Reviewer response for version 2

**DOI:** 10.5256/f1000research.24799.r60388

**Published:** 2020-02-24

**Authors:** Paul Campbell

**Affiliations:** 1 Keele University, Keele, UK; 2 Midlands Partnership NHS Foundation Trust, Stafford, UK

The authors have successfully addressed my previous comments and I feel the paper has improved in terms of content and clarity. I have no further comments and wish the team well in their endeavours to development a knee pain based prognostic model for children and adolescents.

Is the study design appropriate for the research question?

Partly

Is the rationale for, and objectives of, the study clearly described?

Yes

Are sufficient details of the methods provided to allow replication by others?

Partly

Are the datasets clearly presented in a useable and accessible format?

Not applicable

Reviewer Expertise:

Psychology, Psychometrics, Epidemiology, Musculoskeletal

I confirm that I have read this submission and believe that I have an appropriate level of expertise to confirm that it is of an acceptable scientific standard.

# Peer review: Reviewer response for version 1

**DOI:** 10.5256/f1000research.23966.r59038

**Published:** 2020-01-27

**Authors:** Paul Campbell

**Affiliations:** 1 Keele University, Keele, UK; 2 Midlands Partnership NHS Foundation Trust, Stafford, UK

Many thanks for the opportunity to review this protocol paper that reports on the rationale and methodology of testing an adolescent knee pain prognostic tool. The protocol is generally well written, and the proposed project to develop a prognostic tool is much needed within the child and adolescent musculoskeletal community. However I do feel some points within the paper require greater clarity and have set out a number of recommendations that I feel would improve the content and presentation of this paper.

1. Whilst the authors have aptly discussed the rationale for a prognostic tool within the introduction and briefly touch upon stratified care, I feel they need also mention what this means in terms of aligned treatments based on the risk (i.e. the pathway for stratified patients), a little bit more on this would benefit the introduction.
2. In the exclusion criteria the authors mention participants will be excluded if they have an inability to understand the questionnaire, is this a language issue, or for those with learning difficulties, or both, I think the authors might wish to expand on this a little more to give examples.
3. The authors state that both parents will be approached before the consultation, does that then require that both parents be present as this won’t often be the case (parent working, single parent etc), also more fundamental is the issue of “before the consultation”, if this is the case how do they know that the knee pain is not referred pain from the spine for example or that whilst the child/adolescent has knee pain that they may also discuss other issues within the consultation and the focus is on those other issues? I might add that the child may be looked after by someone who is not the parent (family member) and perhaps the term “caregiver” should also be used when the term parent is used?
4. The authors describe complementary recruitment strategies to work alongside primary care recruitment, I think the use of wider recruitment strategy is good considering the low participation rate of children within primary care to MSK studies. However, whilst the authors have stipulated that children/adolescents will be reminded of the premise for participation, i.e. that participants have been seen by their GP for knee pain, how will this be confirmed? Furthermore, the authors have not explained the timeline for this, for example the recruitment within GP practices is at the time of consultation, however social media recruitment does not state a criteria of when they should have consulted. One of the key prognostic markers for adult MSK pain is time since onset, here you may have potentially two distinct groups, one who have consulted (not clear whether this will be the first consultation or a subsequent visit), and those via social media at presumably post consultation (but not stipulated as to what time period criteria is acceptable, is it a week, month, year etc), this presents issues of case mix, and at the very least the authors would have to collect information about timing of consultations (and timing of when patient first experienced pain) to factor into their analysis.
5. I think the offer of cinema tickets is a good incentive, however would it not be better to offer a family ticket or at least two tickets as a child would most likely go on their own?
6. For the data collection section, I wonder if a diagram might be helpful here as it is not fully clear, does the GP collect the data post consultation, will they (GPs) have time to do this, the authors mention previously that the child/adolescent will be approached to complete the tool before consultation, so does this mean they fill in the tool before consultation and then other information post consultation? The authors should be aware that pre-consultation may reveal different responses to a post-consultation (e.g. reductions in fear avoidance for parent/child due to reassurance from GP etc), this point also relates to point 4 above where alternative recruitment strategies may be used that could lead to different responses due to different timings?
7. When discussing the outcomes, the authors describe that outcomes will be collected by a combination of self-report or parent report, firstly this doesn’t make sense because of the inclusion of “or” as that implies either, whereas it is described as a combination. Does this mean that there will be a choice in who fills in the questionnaire, should it not be self-report AND parent report, or is this related to age and access to online social media platforms where parents/caregivers would have to complete, I think this needs to be explained, and again the researchers should collect information on who fills in the questionnaire as there are differences between proxy and self-report.
8. Participant timeline, is table 1 needed, I think it is clear that there will be an assessment at baseline and follow up at 3 months and 6 months for each participant, I don't think a diagram is needed for that? I feel a recruitment flow diagram would be a much better inclusion for this paper.
9. The authors discuss the development of the items for the tool, I think a little more should be said about this, for example is this the information within the review (ref 31) or additional literature, also as this is a generation of the candidate items for a prognostic tool it would be good to give some detail (e.g. how many studies within the review, what sort of populations, were they all prospective studies etc). In addition the authors discuss the situation where multiple items or multiple scales were present for items, I feel this whole sentence could do with expansion, what is meant by multiple items and multiple scales, do you mean prognostic factors that have been measured suing multiple items/scales? Give some examples and how a multiple scale construct is then reduced to a single question, how was this done?
10. Within the study piloting section, the authors mention the process of improving face, construct, content validity, stressing the importance of this should a GP make an inaccurate diagnosis, I have some concern about this statement as it suggests that the questions asked about pain via the tool are in some way involved in diagnosis, this is surely not the case, can the authors explain what they mean?
11. In the stability of the tool section the authors state that children were referred to primary care because of their knee pain, who is it then who refers children to primary care, what is the process for this as this may be a factor to be considered (e.g. how long does it take for a person to be referred to primary care, who does the referral, how are decisions made on a referral etc), I thought it was that the child/parent/caregiver attends primary care?
12. I am slightly confused about the test-retest parameters, from what is written it appears that the test was done pre and post consultation (i.e. within the same day, perhaps within the same hour), this timeline is too short, the point of test/retest is to ensure that the participants would not remember the responses to the questions asked, so for example the next week, to then ensure that the responses are consistent, I am not convinced this current test stacks up, and any differences would likely be via information received within the consultation (influencing different responses) rather than the actual difference coming from inconsistency in the measure (for example a GP offering reassuring information).
13. I feel for the general readership it would be helpful to have the tool in English within the paper, and when I tracked down the English version I could see it has 12 questions, whereas the one in the current paper has 13 questions?
14. The secondary outcome is the same as the primary outcome, with the only difference being time, could the authors clarify whether this is actually a secondary outcome or just a different time period for the same primary outcome measure?
15. The authors state that the statistical analysis plan can be found elsewhere, this protocol paper should include the full statistical analysis plan rather than have a signpost to another protocol, can the authors include the full details in this paper (and if they have then they don’t need to reference another protocol).
16. The authors discuss potential floor and ceiling effects, can the authors say a little more about this as presumably some risk items would only identify those at extremes (i.e. at risk) in any case? This is particularly true of items that can identify those at high risk, they may only account for a small proportion of the population with a number of items giving an additive high risk score?
17. Will the authors examine both each individual item but also within a model (multivariable) to test the independent effects of each item as some many have overlapping qualities (will they also examine redundant items); also will the authors consider model fit (e.g. explained variance) within their analysis?
18. Point 5 in the statistical plan is not explained well, could the authors say a little more about the identification of cut off scores and what criteria will be used to decide on this (i.e. are these independently generated from other sample or this one, what determines a poor outcome, if determined from this cohort it will make external validity more problematic), also if more will be given to sensitivity of the tool, how will this factor in the development of risk groups? Also it might be beneficial for the authors to compare risk groups in terms of the baseline characteristics and test across those to give further discriminant information, and in addition it might be worth considering construct validity by comparing to any existing measures, for example this measure could be compared to the generic PPST?
19. The discussion might benefit more by describing the potential for the tool in terms of the assistance to clinicians, for example within the study will the research consider how clinicians feel about the tool and whether they feel it would be useful, also what is not clear at present is the potential pathways that are available as defined by risk, do the proposed risk groups have an aligned pathway already for example, if so what might that look like and if not how will these be developed?

Is the study design appropriate for the research question?

Partly

Is the rationale for, and objectives of, the study clearly described?

Yes

Are sufficient details of the methods provided to allow replication by others?

Partly

Are the datasets clearly presented in a useable and accessible format?

Not applicable

Reviewer Expertise:

Psychology, Psychometrics, Epidemiology, Musculoskeletal

I confirm that I have read this submission and believe that I have an appropriate level of expertise to confirm that it is of an acceptable scientific standard, however I have significant reservations, as outlined above.

# Response

**Published:** 2020-02-15

**Authors:** Alessandro Andreucci

**Affiliations:** Center for General Practice at Aalborg University, Denmark

Dear Editorial Team, F1000 Research,

We were delighted that the Reviewers of our recent submission " The Adolescent Knee Pain (AK-Pain) prognostic tool: protocol for a prospective cohort study" (ID: 21740) provided us with their very helpful feedback on our paper. This has given us the opportunity to apply changes that have led to an improvement of the paper.

**Reviewer 1**

Many thanks for the opportunity to review this protocol paper that reports on the rationale and methodology of testing an adolescent knee pain prognostic tool. The protocol is generally well written, and the proposed project to develop a prognostic tool is much needed within the child and adolescent musculoskeletal community. However I do feel some points within the paper require greater clarity and have set out a number of recommendations that I feel would improve the content and presentation of this paper.

Response: We thank the reviewer for the valuable feedback provided. We have addressed the comments and changed the manuscript (changes are underlined in the text), which we feel is now improved. Individual responses for each specific comment are outlined below.

1.     Whilst the authors have aptly discussed the rationale for a prognostic tool within the introduction and briefly touch upon stratified care, I feel they need also mention what this means in terms of aligned treatments based on the risk (i.e. the pathway for stratified patients), a little bit more on this would benefit the introduction.

Response: We thank the reviewer for the comment and have now amended the introduction by providing more information on the aligned treatments based on the risk.

The text (Introduction section) now reads “One potential option to support clinical decision making is to develop decision aids such as prognostic tools. These tools often consist of items with prognostic value that can be asked during the clinical consultation or completed prior to the consultation. Thereby patients can be stratified depending on their prognosis (19) and subsequently the best targeted treatment based on the prognostic profile can be offered the patient. In the case of a child or adolescent with knee pain, different treatments or recommendations (e.g. short education session, modification of physical activity levels, exercise, use of painkillers, referral to a specialist) might be provided depending on the risk of a poor prognosis. Examples of prognostic tools that have already been developed include the Keele STarT Back Screening Tool (SBST) for lower back pain in adults (20) or the Pediatric Pain Screening Tool (PPST) for general pediatric pain (19).”

2.     In the exclusion criteria the authors mention participants will be excluded if they have an inability to understand the questionnaire, is this a language issue, or for those with learning difficulties, or both, I think the authors might wish to expand on this a little more to give examples.

Response: Participants are meant to be excluded if they have either language issues or learning difficulties.

The text (section Methods, Eligibility and recruitment of participants) now reads “Inability to take part in the study due to an inability to understand the questionnaire (i.e. participants who have issues with Danish language or have learning difficulties)”

3.     The authors state that both parents will be approached before the consultation, does that then require that both parents be present as this won’t often be the case (parent working, single parent etc), also more fundamental is the issue of “before the consultation”, if this is the case how do they know that the knee pain is not referred pain from the spine for example or that whilst the child/adolescent has knee pain that they may also discuss other issues within the consultation and the focus is on those other issues? I might add that the child may be looked after by someone who is not the parent (family member) and perhaps the term “caregiver” should also be used when the term parent is used?

Response: In Denmark, the general practitioner and his/her staff know in advance if the person is consulting for knee pain based on the booking. However, the general practitioner might also be made aware of the knee pain problem during a consultation regarding other issues. In this case, the questionnaire will be filled out after the consultation. As the reviewer correctly pointed out however, the knee pain might be referred from other body sites (e.g. spine), and we acknowledge that this is a limitation of our study. 

We have amended the text and made it clearer. The text (section Methods - Eligibility and recruitment of participants) now reads “Participants will be recruited from general practices on the basis of consultation for knee pain. In Denmark, consultations with the GP are booked beforehand, which allows the GP and the GP’s staff to know in advance what the patient is consulting for. The study will be introduced to the caregivers and children before the consultation by a person working at the general practice (e.g. the GP/nurse/secretary). If patients meet the inclusion criteria for being eligible into the study (e.g. patient aged between 8 and 19 years old who consult for knee pain) and agree to participate, they will be provided with the study material (i.e. prognostic tool to be completed). However, if the GP was made aware of the issue of the presence of knee pain during a consultation regarding other health issues, the questionnaire will be completed after the consultation.”

4.     The authors describe complementary recruitment strategies to work alongside primary care recruitment, I think the use of wider recruitment strategy is good considering the low participation rate of children within primary care to MSK studies. However, whilst the authors have stipulated that children/adolescents will be reminded of the premise for participation, i.e. that participants have been seen by their GP for knee pain, how will this be confirmed? Furthermore, the authors have not explained the timeline for this, for example the recruitment within GP practices is at the time of consultation, however social media recruitment does not state a criteria of when they should have consulted. One of the key prognostic markers for adult MSK pain is time since onset, here you may have potentially two distinct groups, one who have consulted (not clear whether this will be the first consultation or a subsequent visit), and those via social media at presumably post consultation (but not stipulated as to what time period criteria is acceptable, is it a week, month, year etc), this presents issues of case mix, and at the very least the authors would have to collect information about timing of consultations (and timing of when patient first experienced pain) to factor into their analysis.

Response: We thank the reviewer for the very insightful comment.

Our plan is to include only those who have consulted within one week. In addition, sensitivity analysis will be applied by comparing results of those recruited in primary care and those recruited through social media to assess potential differences related to case mix. Regarding timing of when patient first experienced pain, this is already assessed with item number 4 of the prognostic tool.

We have amended the text and made it clearer. The text (section Methods - Eligibility and recruitment of participants) now reads “Complementary recruitment strategies that might be applied to maximize the recruitment rate are the advertisement of the study through social media (e.g. Facebook, Twitter, Snapchat) and through explicative posters displayed at the participating general practices. In this case, it will be screened that participants have been seen by their GP within the last week and knee pain was part of the consultation.”

5.     I think the offer of cinema tickets is a good incentive, however would it not be better to offer a family ticket or at least two tickets as a child would most likely go on their own?

Response: Although the idea of offering a family ticket or at least two tickets is good, unfortunately we were limited in our choice by financial constrictions. This is why we can offer only one cinema ticket at baseline and one cinema ticket at follow-up, and unfortunately it is not possible to change this strategy now as the study is already on-going.

6.     For the data collection section, I wonder if a diagram might be helpful here as it is not fully clear, does the GP collect the data post consultation, will they (GPs) have time to do this, the authors mention previously that the child/adolescent will be approached to complete the tool before consultation, so does this mean they fill in the tool before consultation and then other information post consultation? The authors should be aware that pre-consultation may reveal different responses to a post-consultation (e.g. reductions in fear avoidance for parent/child due to reassurance from GP etc), this point also relates to point 4 above where alternative recruitment strategies may be used that could lead to different responses due to different timings?

Response: We thank the reviewer for the very insightful comment.

Data collection will occur before the consultation and will be facilitated by the general practitioner’s staff (e.g. nurse, secretary), with the tool to be fully completed before consultation. 

However, as pointed out at point 3 above, the knee pain problem might not be cited as the primary reason for consultation, and the GP only made aware of it during the consultation. In this case, the tool will be completed after the consultation. Being aware of the potential differences in parameters pre-consultation vs. post-consultation we assessed the stability of the tool, which showed good stability of the tool parameters before and after consultation with the general practitioner, as described in section “Methods – Stability of the tool”.

7.     When discussing the outcomes, the authors describe that outcomes will be collected by a combination of self-report or parent report, firstly this doesn’t make sense because of the inclusion of “or” as that implies either, whereas it is described as a combination. Does this mean that there will be a choice in who fills in the questionnaire, should it not be self-report AND parent report, or is this related to age and access to online social media platforms where parents/caregivers would have to complete, I think this needs to be explained, and again the researchers should collect information on who fills in the questionnaire as there are differences between proxy and self-report.

Response: We thank the reviewer for the comment. The outcome will be collected by questionnaires that will be sent through an e-mail with a link to the web application for online surveys or text message. Within the questionnaire, there are also questions about name, surname and contact information (e-mail and phone number) and about who the person replying to the questionnaire is (i.e. child alone/caregiver/child together with the caregiver). This allows us to know if the person replying is the caregiver or the child/adolescent.

We have amended the text and made it clearer. The text (section Methods – Data collection) now reads: “ Outcomes will be collected by questionnaires. The adolescent can choose to do this self-reported or caregiver-reported (through an e-mail with a link to the web application for online surveys or text message) at follow-up (two time points: 3-month and 6-month follow-up). The questionnaire also includes a question about who the person replying to the questionnaire is (i.e. child alone/caregiver/child together with the caregiver)”

8.     Participant timeline, is table 1 needed, I think it is clear that there will be an assessment at baseline and follow up at 3 months and 6 months for each participant, I don't think a diagram is needed for that? I feel a recruitment flow diagram would be a much better inclusion for this paper.

Response: We have replaced table 1 with a recruitment flow diagram and modified the text, which now reads “ The process of recruitment and retainment of participants in the study (data collection start date: July 2019 – end date: June 2020) is described in the following flow diagram (figure 1).”

9.     The authors discuss the development of the items for the tool, I think a little more should be said about this, for example is this the information within the review (ref 31) or additional literature, also as this is a generation of the candidate items for a prognostic tool it would be good to give some detail (e.g. how many studies within the review, what sort of populations, were they all prospective studies etc). In addition the authors discuss the situation where multiple items or multiple scales were present for items, I feel this whole sentence could do with expansion, what is meant by multiple items and multiple scales, do you mean prognostic factors that have been measured suing multiple items/scales? Give some examples and how a multiple scale construct is then reduced to a single question, how was this done?

Response: We thank the reviewer for the insightful comment.

Items to be included in the prognostic tool were selected based on a combination of those initially identified with the review (31), of the wider literature on child and adolescent musculoskeletal pain and from meta-analysis of individual participant data (PROSPERO ID CRD42019116861). This allowed us to include also other potential factors that were not previously assessed in the studies identified in the review but were found to be associated with the prognosis of general musculoskeletal pain in other studies and might therefore result as significant contributors to the prognostic model. Regarding the situation where multiple items or multiple scales were present for items relative to the prognostic factors, single items were selected from the most appropriate scales.

We have now modified the text (Section Methods - Development of the tool) and made it clearer. The text now reads: 

“Prognostic factors for knee pain in children and adolescents that can be measured in the context of general practice were identified from a review of the current literature on the topic (29). The review included 26 prospective studies, of which 4 focused on knee pain. These 4 studies included schoolchildren 12-19 years old from Denmark (3 studies) and 10-12 years old from Finland. Initially, the most important domains for the prognosis of knee pain and the specific items to be included in the tool were selected based on the review but also from strength of association from previous studies identified within the literature and from meta-analysis of individual participant data. Prognostic factors for knee pain identified within the review (29) were increasing age, pain frequency, practicing sport more than 2 times/week and low quality of life (second question of item 9 within the tool). Other factors that were identified from the wider literature and meta-analysis of individual participant data (PROSPERO ID CRD42019116861) were knee pain characteristics (pain duration, traumatic/non-traumatic pain onset, limitations in daily activities due to knee pain, presence of knee pain in one knee or both knees ), presence of pain in other body sites, gender, sleep, smoking, psychological factors, parental history of pain. During this stage, great emphasis was given to include only the most necessary factors considering the ultimate use of the prognostic tool (i.e. it should be used in a reasonable time of a consultation within general practice). Items relative to the specific prognostic factors were initially selected from validated scales when possible (e.g. regarding pain characteristics, limitation in daily activities, sport participation, psychological factors, sleep), or from previous studies. When multiple items within a scale or multiple scales were available for a prognostic factor, the relevant literature on the topic was identified and discussed at meetings with GPs and staff working at the Center of General Practice at Aalborg University in order to select the most appropriate items within the possible options. For example, the sleep item was selected from the self-reported Pediatric Quality of Life Inventory, one question of the psychological factor item from the Pain Catastrophizing Scale for Children, one question of the psychological factor item and the item relative to limitations in daily activities from the EuroQol Five-Dimensional Questionnaire Youth version.”

10.  Within the study piloting section, the authors mention the process of improving face, construct, content validity, stressing the importance of this should a GP make an inaccurate diagnosis, I have some concern about this statement as it suggests that the questions asked about pain via the tool are in some way involved in diagnosis, this is surely not the case, can the authors explain what they mean?

Response: The aim of the study piloting was to improve the comprehensibility and wording of the questions included in the tool, so that adolescents would provide information about the parameters related to the knee pain prognosis that were as close as possible to the real values and therefore limit potential misreporting. As the reviewer correctly pointed out, the tool is not conceived as a replacement of the diagnosis made by the general practitioner, but only as a support for understanding the prognosis together with the clinical examination.

We have now modified the text and made it clearer. The text (section Methods – Study piloting) now reads: 

“The aim was to improve the face, construct and content validity of the tool at this stage. This is especially important considering that a worse outcome can result if there is a lack of communication between the GP and the child about the knee pain characteristics and the factors related to knee pain assessed within the tool (33).”

11.  In the stability of the tool section the authors state that children were referred to primary care because of their knee pain, who is it then who refers children to primary care, what is the process for this as this may be a factor to be considered (e.g. how long does it take for a person to be referred to primary care, who does the referral, how are decisions made on a referral etc), I thought it was that the child/parent/caregiver attends primary care?

Response: We have amended the imprecision in the text, as we actually meant the child/adolescent who consult primary care for knee pain. We have modified the text, which now reads “ Children and adolescents who consulted primary care and their caregivers were given the prognostic tool (together with the informed consent) to be completed in the waiting room of the general practice before the consultation.”

12.  I am slightly confused about the test-retest parameters, from what is written it appears that the test was done pre and post consultation (i.e. within the same day, perhaps within the same hour), this timeline is too short, the point of test/retest is to ensure that the participants would not remember the responses to the questions asked, so for example the next week, to then ensure that the responses are consistent, I am not convinced this current test stacks up, and any differences would likely be via information received within the consultation (influencing different responses) rather than the actual difference coming from inconsistency in the measure (for example a GP offering reassuring information).

Response: We thank the reviewer for the comment and acknowledge the inaccuracy in the language used within the text. As pointed out by the reviewer, the GP might influence the perception of certain parameters related to pain (i.e. psychological factors, pain duration) through the discussion occurring during the consultation. In order to test if responses to the questions asked were influenced by the consultation with the GP (and to estimate the difference in responses), the stability of the tool before and after the consultation was tested and results showed good stability. However, as the reviewer correctly pointed out, this cannot be considered a test-retest as the time frame between the two measurements was too short. We have amended the text, (Section Methods – Stability of the tool) which now reads “ A pilot study to assess the stability of the prognostic tool (31) in children and adolescents pre- and post- consultation was carried out.”

13.  I feel for the general readership it would be helpful to have the tool in English within the paper, and when I tracked down the English version I could see it has 12 questions, whereas the one in the current paper has 13 questions?

Response: The English version, with 13 questions is accessible at: https://doi.org/10.7910/DVN/QKWOOT 

14.  The secondary outcome is the same as the primary outcome, with the only difference being time, could the authors clarify whether this is actually a secondary outcome or just a different time period for the same primary outcome measure?

Response: We thank the reviewer for the comment and have amended the imprecision in the text. In our study, the outcome is the same is collected at two endpoints. We decided to consider the knee pain prognosis at 3-month as the primary endpoint and the knee pain prognosis at 6 months as a secondary endpoint.

We have modified the text (Section Methods – Outcome), which now reads “ The outcome measure will be the recurrence/persistence of activity-limiting knee pain (i.e. defined as participants reporting yes to having pain that is limiting activities in the same knee) at follow-up (28). Participants will be asked about continuity of their knee pain (i.e. “do you still have knee pain?” and, if they reply “no”, “when did your stop having knee pain?”), to enable the distinction between recurrence (on/off knee pain episodes between baseline and follow-up) and persistence (continuous knee pain from baseline to follow-up) of knee pain. The primary end-point that will be collected is the recurrence/persistence of activity-limiting knee pain at 3-month follow-up, while the secondary end-point is the recurrence/persistence of activity-limiting knee pain at 6-month follow-up”

15.  The authors state that the statistical analysis plan can be found elsewhere, this protocol paper should include the full statistical analysis plan rather than have a signpost to another protocol, can the authors include the full details in this paper (and if they have then they don’t need to reference another protocol).

Response: We have now corrected the text (section Methods – Statistics), inserted the full analysis plan within this paper and deleted the reference to the other protocol.

16.  The authors discuss potential floor and ceiling effects, can the authors say a little more about this as presumably some risk items would only identify those at extremes (i.e. at risk) in any case? This is particularly true of items that can identify those at high risk, they may only account for a small proportion of the population with a number of items giving an additive high risk score?

Response: We thank the reviewer for the very insightful comment. As correctly pointed out by the reviewer, for items that represent an ordinal or categorical variable with more than two potential response categories (for example 5 categories), the responses given might be only the top or bottom extreme of the scales. For example, this might happen for items related to psychological factors and sleep. In this case, participants with higher levels of anxiety/depression, helplessness or sleep problems would most likely fall in the high-risk category. A potential option to account for this is to initially test the independent effect of each item by means of multiple logistic regression, include those items that show a significant contribution to the model and subsequently apply different weights based on the strength of association. For example, a combination of the different items can be used to identify the scores for defining the risk groups (i.e. low/medium/high) and if items that identify only a small proportion of the population (e.g. high levels of psychological factors or sleep problems) are associated with a high risk of a bad prognosis, they can be selected and used to give an additive high-risk score.

17.  Will the authors examine both each individual item but also within a model (multivariable) to test the independent effects of each item as some many have overlapping qualities (will they also examine redundant items); also will the authors consider model fit (e.g. explained variance) within their analysis?

Response: We thank the reviewer for the comment. The plan is to test the independent effect of each item by means of multiple logistic regression and include only those items that show a significant contribution to the model. Therefore, the items that will not show a significant contribution will be excluded. In addition, items might be weighted differently based on the strength of association.

We have modified the text (section Methods – Statistics), which now reads: “ We will estimate the knee pain prognosis(i.e. recurrence/persistence of knee pain, dichotomous outcome) at 3-month and 6-month follow-up by means of multiple logistic regression to estimate ORs and 95% confidence intervals for each item included in the tool. This allows to assess the independent effects of each item and will inform on which factors are most related to the prognosis of knee pain ( only the items that will show a statistically significant contribution to the model will be selected ). This will also provide an insight on the scores of the prognostic tool (both overall and for subscales) to be applied for the creation of the initial risk groups. Alternatively, the RR will be estimated if another linear model analysis will be carried out. In addition, a potential option is to apply different weights to the items based on the strength of association.”

18.  Point 5 in the statistical plan is not explained well, could the authors say a little more about the identification of cut off scores and what criteria will be used to decide on this (i.e. are these independently generated from other sample or this one, what determines a poor outcome, if determined from this cohort it will make external validity more problematic), also if more will be given to sensitivity of the tool, how will this factor in the development of risk groups? Also it might be beneficial for the authors to compare risk groups in terms of the baseline characteristics and test across those to give further discriminant information, and in addition it might be worth considering construct validity by comparing to any existing measures, for example this measure could be compared to the generic PPST?

Response: We thank the reviewer for the insightful comment and provide some clarifications on this point.

At each follow-up time-point, a poor outcome is defined as the presence of activity-limiting knee pain. As the cut-off scores will be generated by using data of this sample, the prognostic model developed within this cohort will have to be externally validated with another cohort in the future. Regarding sensitivity, we stated that more importance will be given to sensitivity of the tool, meaning that we will choose cut-off scores for the definition of risk groups that will allow to identify the majority of those with a potential bad prognosis in the high risk group, and rule out from the group those with a good prognosis. 

In addition, despite comparing the prognostic tool to the pediatric pain screening tool might be an option, it should be taken into account that the pediatric pain screening tool was validated in a tertiary care clinic and may operate differently in a primary care setting, which is where our prognostic tool is meant to operate. Therefore, the comparison might result in a poor match between risk group categories of the two tools due to differences in the severity of the sample used to develop the tools.

We have modified the text, which now reads: “ Data from this sample will be used for the creation of risk groups for the recurrence/persistence of knee pain, on the basis of cut-off scores identified using the ROC curves. Weights based on the strength of association identified with the multiple logistic regression might be applied. The initial idea is to have two or three risk groups (e.g. low/medium/high), which have to be clinically meaningful. More importance will be given to the sensitivity of the tool over the specificity. This means that in the presence of different cut-off scores for the inclusion of patients in the high-risk group, the cut-off that will allow to identify the majority of those with a bad prognostic outcome will be chosen. This will be done to avoid the misclassification of patients at high-risk in the medium or low-risk group.”

19.  The discussion might benefit more by describing the potential for the tool in terms of the assistance to clinicians, for example within the study will the research consider how clinicians feel about the tool and whether they feel it would be useful, also what is not clear at present is the potential pathways that are available as defined by risk, do the proposed risk groups have an aligned pathway already for example, if so what might that look like and if not how will these be developed?

Response: We thank the reviewer for the insightful comment and provide some clarifications on this point.

There are other steps that will follow the development of this tool. As the reviewer highlights, there are additional steps to be completed before implementing the tool. This includes 1) an external validation of the tool 2) developing the matched care pathways 3) mixed-methods studies to understand how the tool should look and operate in the context of general practice, and 4) subsequent clinical trials to test the impact of the tool. A cluster randomized controlled trial will be performed where patients will be randomized to stratified care provided by using the tool or to the best current care, and differences in clinical outcomes will be assessed. The original idea is to provide targeted treatments to the three risk groups. For example, patients in the low risk group might be provided with an initial short patient education session and given a leaflet that reinforces the information given by the GP. For the medium risk group, strengthening exercises and load management might be delivered, while for the high-risk group a tailored treatment might be delivered that will target also psychological factors. However, these are only initial suggested treatments, and additional treatments might be developed depending on the items that will prove to be significantly associated with a bad prognosis (e.g. potential further treatments might be advices regarding having a good sleep hygiene). Regarding assistance to the clinician, this tool together with another tool for the knee pain diagnosis, will be part of a package (decision tool) that will assist clinicians in the diagnosis and treatment of knee pain. Future qualitative studies will be needed to assess the GPs´ behaviour when using the tool.

We have now modified the text and added this part to the section "Use of the tool for providing stratified care":

“The development of this tool fits within a wider research program, which overall aim is to provide a stratified approach to primary care management of child and adolescent knee pain that can result in clinical and economic benefits compared with current best practice. Therefore, future perspectives include the use of this tool in a randomized controlled trial, which will investigate whether subgrouping patients using the tool, combined with targeted treatment, is more clinically effective (i.e. it will reduce long- term disability from knee pain) and cost-effective compared to best current care. In addition, there is scope for performing future qualitative studies to assess the GPs´ behavioral change when using the tool (e.g. changes in referral to physical therapy, diagnostic tests and medication prescriptions)”

**Reviewer 2**

Thank you for giving me the opportunity to review this well described paper about development and test of a prognostic tool for knee pain in adolescence. A very important area. I am very pleased to see the incorporation of psychosocial aspect in the pain.

I was wondering about the use of the word Ængstelig/deprimeret (anxious/depressed). Do children aged 8 and a bit older understand that word? Would it be better to use the word bekymret (worried) instead? 

In the statistics section; you write that you will stratify by traumatic/non-traumatic onset. Why do they choose to do so? 

I think this paper is well written and described and I therefore have no further comments. I am looking forward the seeing the results.

Response: We thank the reviewer for the valuable feedback provided. We have addressed the comments, and individual responses for each specific comment are outlined below.

 

1. I was wondering about the use of the word Ængstelig/deprimeret (anxious/depressed). Do children aged 8 and a bit older understand that word? Would it be better to use the word bekymret (worried) instead?

 

We thank the reviewer for the insightful comment.

The item used to ask about the psychological status was taken from the validated Danish version of the EQ-5d. However, because of the same concern regarding understanding the word Ængstelig/deprimeret, we asked children and adolescents during the cognitive interviews if they had any problems with this word. As it was difficult for some of the younger children to understand this word, we decided to add a note explaining what Ængstelig/deprimeret means. The note was “Ængstelig/deprimeret svarer til at være ked af det (det handler om hvordan du har det, og ikke nødvendigvis på grund af dine smerter)” (In English, anxious/depressed corresponds to be sad – it’s about how you feel and not necessarily because of your knee pain). After adding the note, children did not have further problems with the word.

 

1. In the statistics section; you write that you will stratify by traumatic/non-traumatic onset. Why do they choose to do so? 

 

We thank the reviewer for the insightful comment.

The reason why we decided to stratify analysis by traumatic/non-traumatic onset is that previous research has indicated clear differences in the prognosis depending on the type of knee pain onset in both children and adults (please see references below). 

If the stratification will provide very different prognostic results, it might be possible to use this factor for a quick initial discrimination between low risk vs. medium/high-risk of a bad prognosis.

References:

El-Metwally A. Lower Limb Pain in a Preadolescent Population: Prognosis and Risk Factors for Chronicity--A Prospective 1- and 4-Year Follow-up Study. Pediatrics. 2005;116(3):673-681. doi:10.1542/peds.2004-1758

Belo JN, Berger MY, Koes BW, Bierma-Zeinstra SMA. Prognostic factors in adults with knee pain in general practice. Arthritis Care Res. 2009;61(2):143-151. doi:10.1002/art.2441

# Peer review: Reviewer response for version 1

**DOI:** 10.5256/f1000research.23966.r58102

**Published:** 2020-01-27

**Authors:** Signe Fuglkjær Møller

**Affiliations:** 1 Hartvigsen and Hein Back Centre and Sports Clinic, Odense, Denmark

Thank you for giving me the opportunity to review this well described paper about development and test of a prognostic tool for knee pain in adolescence. A very important area. I am very pleased to see the incorporation of psykosocial aspect in the pain.

 I was wondering about the use of the word Ængstelig/deprimeret (anxious/depressed). Do children aged 8 and a bit older understand that word? Would it be better to use the word bekymret (worried) instead? 

 In the statistics section; you write that you will stratify by traumatic/non-traumatic onset. Why do they choose to do so? 

 I think this paper is well written and described and I therefore have no further comments. I am looking forward the seeing the results.

Is the study design appropriate for the research question?

Yes

Is the rationale for, and objectives of, the study clearly described?

Yes

Are sufficient details of the methods provided to allow replication by others?

Yes

Are the datasets clearly presented in a useable and accessible format?

Yes

Reviewer Expertise:

musculoskeletal health

I confirm that I have read this submission and believe that I have an appropriate level of expertise to confirm that it is of an acceptable scientific standard.
